# Nosso Precioso Escore de Cálcio

**DOI:** 10.36660/abc.20200854

**Published:** 2020-09-18

**Authors:** Henrique Trad

**Affiliations:** 1 Lotus Radiologia Ribeirão Preto SP Brasil Lotus Radiologia, Ribeirão Preto, SP - Brasil

**Keywords:** Doença da Artéria Coronariana, Placa Aterosclerótica, Calcificação Vascular, Rigidez Vascular, Tomografia Computadorizada por Raios X/métodos, Cálcio, Pontuação de Propensão

Algumas coisas são fortes, robustas. Nos exames de imagem em geral, essas coisas se baseiam em princípios sólidos da física que podem resistir às iniquidades do uso diário. Na tomografia computadorizada (TC), as principais densidades teciduais são sólidas, medidas em unidades Hounsfield (HU), e algumas são realmente fortes, fortes em cálcio, por assim dizer. A resolução de contraste é uma questão a ser considerada nas imagens de TC em geral, principalmente na ampla gama de estruturas encontradas no espectro de densidade da água. Mas as densidades em ambas as extremidades de todo o espectro HU, ou seja, ar e gordura de um lado e osso e metal do outro, são representadas claramente. Além disso, elas poderiam ser medidas com grande precisão e, como essas imagens são de origem matemática, há muito mais do que o olho pode ver que pode ser medido em uma imagem de TC, e há várias medições que podem ser feitas em exames bastante simples. Mas vamos voltar a esse assunto depois.

A medição da quantidade de calcificação nas artérias coronárias é algo bastante antigo.^1^Chama-se escore coronariano de cálcio (ECC). Foi desenvolvido, testado e implementado, teve seu valor clínico demonstrado, mas ainda carece de consenso sobre sua utilização e cobertura de seguro em procedimentos médicos mundo afora.^2-4^ A princípio, a medição tinha que ser feita na tomografia por feixe de elétrons e depois, com a nova geração de TCs. Nesta edição dos Arquivos Brasileiros de Cardiologia, Souza et al.^5^ mostram elegantemente como essa medida de ECC tem excelente correlação, feita da maneira “usual”, bem como a partir de uma TC de tórax padrão. Isso já foi demonstrado, em diversos exames e com boa precisão, alguns comparando-se com métodos semiquantitativos e visuais, com o consenso da Sociedade de Tomografia Computadorizada Cardiovascular^5^ incluindo um excelente trabalho recente deste mesmo grupo de pesquisadores.^6^ Mas agora, fazendo uma leitura dos números, a correlação é excelente, como já demonstrado em outros estudos,^7^ e a sugestão é que poderíamos medir com precisão o ECC, esta importante ferramenta na estratificação de risco da doença arterial coronariana, a partir de um exame bem mais comum.

Agora, vamos tecer algumas considerações técnicas e suas implicações para o último achado de seu estudo, com o qual eu concordo, segundo o qual poderíamos medir o ECC a partir das imagens de TC de tórax padrão como uma alternativa quando a TC cardíaca apropriada não estiver disponível. A forma “usual” de se obter o ECC é com o disparo cardíaco (*cardiac triggering*), modo axial de aquisição e reconstrução com cortes de 2,5 a 3,0 mm de espessura, e esse tipo de aquisição exige equipamentos de TC que contenham o monitor de disparo cardíaco. Não há recomendações sobre o uso de medicamentos para redução da frequência cardíaca, nem requisitos específicos para a rotação do tubo e linhas de detectores.^2^ Equipamentos com 16 ou mais linhas de detectores são mais comuns hoje em dia, mas os disparos cardíacos nem tanto, principalmente após a recomendação de que angiotomografia de coronárias sejam realizadas com no mínimo 64 linhas de detectores. Os fabricantes também oferecem equipamentos com menos linhas, por isso são mais baratos e mais acessíveis, reduzindo principalmente a rotação do tubo para tubos e outros componentes mecânicos mais baratos. Ainda assim, para a maioria dos parâmetros técnicos, a TC de tórax é basicamente a mesma desses e outros equipamentos. Mantendo a resolução espacial da espessura do corte constante em uma TC de tórax, de 1,0 a 1,5 mm, que é rotina nesses aparelhos, que outras implicações existem, principalmente para a resolução temporal, com menor tempo de rotação do tubo e frequências cardíacas não controladas, para a medição do ECC? Na verdade, em Buddoff et al.^7^ os equipamentos utilizados possuíam no mínimo 16 detectores, com diversos protocolos de aquisição.

Consideraremos agora algumas tendências tecnológicas. Voltando ao meu primeiro parágrafo, sobre medições em uma TC de tórax simples. O fato é que o ECC é forte e pode ser medido em tomografias de tórax de rotina com alta precisão. Faremos isso em um futuro próximo, com certeza. Agora veja outros dados que podem ser extraídos de uma imagem de TC. Por exemplo: enfisema pulmonar^8^ e radiômica^9^. Pense nas novas técnicas de inteligência artificial e nos softwares que serão lançados. Finalmente, o ECC é robusto por natureza e também pela sua relevância. Será de grande valia para a cardiologia clínica e será feito rotineiramente em tomografias de tórax mundo afora. Como muitas outras medidas que poderemos produzir com essas imagens. Só não se preocupe em cobrar.
