# Supplementary material for: Nosso Precioso Escore de Cálcio
Source: Arq Bras Cardiol. 2020 Sep 18;115(3):501–2. [Article in Portuguese] doi: 10.36660/abc.20200854 (PMC9363103; doi:10.36660/abc.20200854)
Supplement: Translation

# Translation: Our Precious Calcium Score

**Authors:** Henrique Trad

**Affiliations:** 1 Brazil Lotus Radiologia, Ribeirão Preto, SP - Brazil

**Keywords:** Coronary Artery Disease, Plaque, Atherosclerotic, Vascular Calcification, Vascular Stiffness, Tomography, X-Ray/methods, Calcium, Propensity Score

Some things are strong, robust. In imaging in general, those are based on solid principles of physics that can resist the inequities of daily use. In computed tomography (CT), the main tissue densities are solid, measured in Hounsfield Units (HU), and some are really strong, calcium-strong, so to speak. Contrast resolution is an issue in CT imaging in general, mainly in the wide range of structures inside the water density spectrum. But the densities on both ends of the whole HU spectrum, i.e., air and fat on one side and bone and metal on the other, are depicted clearly. Also, they could be measured with a great deal of accuracy, and, as these images were mathematical in origin, there is a lot more than the eye can see that could be measured on a CT image, and there are several measurements that could be done on rather simple scans. But let’s revise this latter.

Measuring the amount of calcification in coronary arteries is rather old.^1^ It is termed coronary calcium score (CCS). It has been developed, tested and implemented, has had its clinical value shown, but still lacks consensus of use and insurance coverage in medical procedures around the world.^2-4^ At first, it had to be done in electron beam tomography and then, with the new generation of CT scans. In this issue of *Arquivos Brasileiros de Cardiologia*, Souza et al.^5^ elegantly show how this CCS measurement has excellent correlation as done in the “usual” manner as well as from a standard chest CT. This was shown in the past, to a wide variety of scans and with good accuracy, some comparing with semiquantitative and visual methods, with a consensus of the Society of Cardiovascular Computed Tomography,^5^ including a recent great work from this same group of researchers.^6^ But now, crunching the numbers, the correlation is excellent, as has been shown by others before,^7^ and the suggestion is that we could accurately measure CCS, this important tool in coronary artery disease risk stratification, from a rather more common exam.

Let us now address some technical considerations and its implications for the last finding of their study, which I agree with, according to which we could measure CCS from the standard chest CT images as an alternative when appropriate cardiac CT is not available. The “usual” way of achieving a CCS is with cardiac triggering, axial mode of acquisition and 2.5 to 3.0 mm thickness slice reconstruction, and this type of acquisition demands CT equipment that has the cardiac trigger. There are no recommendations on the use of cardiac frequency reduction medications and no specific requirements for tube rotation and detector rows.^2^ Equipment with 16 or more detector rows are nowadays more common, but cardiac triggering not so much, especially after recommendation that coronary CT angiographies be performed with at least 64 detector rows scans. Vendors have also downgraded equipment with fewer rows, so they are cheaper and more accessible, especially reducing tube rotation for cheaper tube and other mechanical components. Yet, for most of the technical parameters, chest CT is basically the same from this equipment and beyond. Keeping the spatial resolution of slice thickness constant on a chest CT, 1.0 to 1.5 mm, which is routine with those scanners, what other implications are there, especially for temporal resolution, with lower tube rotation time and uncontrolled cardiac frequencies, for the measurement of CCS? In fact, in Buddoff et al.,^7^ the equipment used had at least 16 detectors, with a wide variety of acquisition protocols.

Let us now add some technological trends into consideration. Going back to my first paragraph, about measurements on a rather simple chest CT scan. Fact is, CCS is strong, it can be measured on routine chest CT scans with rather high accuracy. We will be doing that in the near future, that’s for sure. Now look at other data that can be extracted from a CT image, e.g. pulmonary emphysema^8^ and radiomics,^9^ for instance. Think of the emerging artificial intelligence and the software applications that are to be released. Finally, CCS is robust in its nature and also in its significance. It will be of great value for clinical cardiology and it will be routinely done on chest CT around the world. Like many other measures, we will be able to produce on those images. Just don’t bother charging.
